# Interaction of antibacterial compounds with RND eﬄux pumps in *Pseudomonas aeruginosa*

**DOI:** 10.3389/fmicb.2015.00660

**Published:** 2015-07-08

**Authors:** Jürg Dreier, Paolo Ruggerone

**Affiliations:** ^1^Basilea Pharmaceutica International Ltd.,Basel, Switzerland; ^2^Dipartimento di Fisica, Università di Cagliari – Cittadella UniversitariaMonserrato, Italy

**Keywords:** eﬄux, multi-drug resistance, bacteria, RND, substrate recognition, *Pseudomonas aeruginsoa*, antibiotic agents, eﬄux pump inhibitors

## Abstract

*Pseudomonas aeruginosa* infections are becoming increasingly difficult to treat due to intrinsic antibiotic resistance and the propensity of this pathogen to accumulate diverse resistance mechanisms. Hyperexpression of eﬄux pumps of the Resistance-Nodulation-Cell Division (RND)-type multidrug eﬄux pumps (e.g., MexAB-OprM), chromosomally encoded by *mex*AB-*opr*M, *mex*CD-*opr*J, *mex*EF-*opr*N, and *mex*XY (-*opr*A) is often detected in clinical isolates and contributes to worrying multi-drug resistance phenotypes. Not all antibiotics are affected to the same extent by the aforementioned RND eﬄux pumps. The impact of eﬄux on antibiotic activity varies not only between different classes of antibiotics but also between members of the same family of antibiotics. Subtle differences in physicochemical features of compound-pump and compound-solvent interactions largely determine how compounds are affected by eﬄux activity. The combination of different high-resolution techniques helps to gain insight into the functioning of these molecular machineries. This review discusses substrate recognition patterns based on experimental evidence and computer simulations with a focus on MexB, the pump subunit of the main RND transporter in *P. aeruginosa*.

## Introduction

Infectious diseases, including bacterial infections, are among the major causes of mortality worldwide (Mason et al., 2003). Infections by multi-drug resistant (MDR) pathogens are especially difficult to treat and are recognized as a major threat (CDC, 2013; Denys and Relich, 2014; Martinez and Baquero, 2014; ECDC, 2015).

The Gram-negative bacterium *Pseudomonas aeruginosa* is frequently involved in healthcare-associated infections like pneumonia, bloodstream infections, urinary tract infections, and surgical site infections (Hidron et al., 2008; Jones et al., 2009; Zhanel et al., 2010). About 8% of nosocomial infections reported to the Centers for Disease Control and Prevention in the US are ascribed to *P. aeruginosa*. 13% of the severe cases in the US are caused by MDR isolates and 14% of European isolates reported between 2005 and 2013 had combined resistance to several antibiotics (CDC, 2013; ECDC, 2015). MDR phenotypes have been described for clinical isolates from various places all over the world (Potron et al., 2015). MDR *P. aeruginosa* isolates are sometimes resistant to nearly all classes of antibiotics and have lost susceptibility toward fluoroquinolones, aminoglycosides, cephalosporins, and carbapenems (Livermore, 2009; Riou et al., 2010; Poole, 2011; Castanheira et al., 2014).

The outer membrane (OM) of Gram-negative bacteria has an asymmetric structure including an outer leaflet made of lipopolysaccharides (LPS), an inner phospholipid leaflet, and porin channels (Kamio and Nikaido, 1976; Nikaido, 2003). Small, hydrophilic compounds can pass the OM by diffusion through the porin channels whereas large and/or hydrophobic compounds have to go through the lipid bilayer (Hancock, 1997; Delcour, 2009). In the *Escherichia coli* OM, there are trimeric porins like OmpF and OmpC present that allow a relatively rapid diffusion of small, hydrophilic substances (Nikaido and Rosenberg, 1983; Cowan et al., 1992; Schulz, 1993). *P. aeruginosa* does not make such trimeric porins but expresses the monomeric porin OprF at a low number with a small opening that only allows slow permeation (Angus et al., 1982; Yoshimura and Nikaido, 1982; Sugawara et al., 2006, 2010). *P. aeruginosa* has also specific channels such as OprD for basic amino acids and peptides, which is the main entry passage of carbapenem antibiotics (Nikaido, 2003). The structure of the OM can be adapted by *P. aeruginosa* to decrease the net negative charge of the LPS in response to cationic peptides such as polymyxin B, which act on the negatively charged LPS (Olaitan et al., 2014). Thus, the OM of *P. aeruginosa* strongly reduces the permeability for most antibiotics and provides an effective and adaptable protection against antibacterial agents (Delcour, 2009; Page, 2012).

Many of the compounds that can pass through the OM are actively transported out of the cell again by eﬄux pumps. The low permeability of the OM combined with such eﬄux pumps results in an effective protection against a wide variety of substances including antibiotics (Kumar and Schweizer, 2005; Fernandez and Hancock, 2012). *P. aeruginosa* PAO1 has 12 eﬄux systems of the Resistance-Nodulation-Cell Division (RND) family (Poole, 2000, 2004, 2005, 2013; Webber and Piddock, 2003; Piddock, 2006; Zechini and Versace, 2009; Fernandez and Hancock, 2012; Nikaido and Pages, 2012; Blair et al., 2014, 2015b; Delmar et al., 2014; Sun et al., 2014), whereof a set of four RND pumps contributes most significantly to antibiotic resistance: MexAB-OprM, MexCD-OprJ, MexEF-OprN, and MexXY-OprM (Fernandez and Hancock, 2012). MexB transports β-lactams including β-lactamase inhibitors and carbapenems (not imipenem), aminoglycosides, fluoroquinolones, tetracyclines, tigecycline, macrolides, amphenicols, novobiocin, sulfonamides, trimethoprim, cerulenin, thiolactomycin, some amphiphilic molecules, disinfectants, dyes, solvents, detergents, and several homoserine lactones involved in quorum sensing [detailed lists of RND substrates are given in (Poole, 2005; Lister et al., 2009) and in **Table 1** for dyes described in this review]. MexD recognizes fluoroquinolones, zwitterionic cephalosporins, macrolides, chloramphenicol, trimethoprim, and tetracyclines. MexF accepts fluoroquinolones, chloramphenicol, trimethoprim, and tetracycline as substrates. MexY transports aminoglycosides, fluoroquinolones, macrolides, tetracyclines, tigecycline, and zwitterionic cephalosporins (Morita et al., 2012).

**Table 1:** Properties of eﬄux-pump substrates and inhibitors.

Compound	logP (o/w)^a^	TPSA (Å^2^)^b^	MW (Da)^c^	PC+^d^	PC-^e^	Net PC^f^	Properties
**Inhibitors**				
D13-9001	0.85	210.8	693.8	9.96	-9.96	0	Not transported
PAßN	2.65	149.5	448.6	9.99	-7.99	2	Transported
Mefloquin	4.27	49.7	379.3	6.13	-5.13	1	Quinolone
**Substrates acting as inhibitor^**g**^**				
Minocycline	-0.28	164.6	457.5	7.11	-7.11	0	Antibiotic
Trimethoprim	0.94	106.8	291.3	5.27	-4.28	1	Antibiotic
Taurocholate	2.33	141.0	514.7	4.79	-5.79	-1	Bile acid
Erythromycin	2.76	195.1	734.9	9.73	-8.73	1	Antibiotic
Glycocholate	3.15	129.9	464.6	4.14	-5.14	-1	Bile acid
**Substrates**				
Ceftobiprole	-1.29	213.7	533.6	9.53	-10.5	-1	Antibiotic
Aztreonam	-1.03	201.3	433.4	7.09	-9.10	-2	Antibiotic
Flomoxef	-0.96	171.8	495.5	7.34	-8.34	-1	Antibiotic
Cefazolin	-0.73	158.9	453.5	5.65	-6.65	-1	Antibiotic
Meropenem	-0.47	117.6	383.5	6.09	-6.09	0	Antibiotic
FDG	-0.40	234.3	656.6	10.38	-10.4	0	Intracellular conversion into a fluorescent product
Moxalactam	-0.38	212.0	518.5	7.60	-9.60	-2	Antibiotic
Tigecycline	-0.26	210.3	586.7	9.99	-8.99	1	Antibiotic
Cefamandole	-0.18	153.4	461.5	6.47	-7.47	-1	Antibiotic
(+)-Cerulenin	0.10	72.7	223.3	3.39	-3.39	0	Antibiotic
Cefsulodine	0.21	187.7	531.5	9.08	-10.1	-1	Antibiotic
Gemifloxacin	0.36	125.8	389.4	6.80	-6.81	0	Antibiotic
Doxorubicin	0.51	207.7	544.5	8.89	-7.89	1	Anticancer drug, quenched intracellular fluorescence
Cephalotin	0.58	115.8	395.4	5.48	-6.48	-1	Antibiotic
Sulbenicillin	0.59	140.8	412.4	5.97	-7.97	-2	Antibiotic
Dodecyl- α-D-maltoside	0.70	178.5	510.6	7.00	-7.00	0	Detergent
Levofloxacin	0.80	76.15	360.4	4.57	-5.58	-1	Antibiotic
Norfloxacin	0.85	80.3	319.3	5.46	-5.46	0	Antibiotic
Nalidixic acid	0.88	73.3	231.2	3.14	-4.15	-1	Antibiotic
Chloramphenicol	1.10	115.4	323.1	5.02	-5.02	0	Antibiotic
Ciprofloxacin	1.16	80.3	331.3	5.66	-5.66	0	Antibiotic
Oxolinic acid	1.19	78.9	260.2	3.25	-4.25	-1	Antibiotic
Carbenicillin	1.23	129.7	376.4	5.32	-7.32	-2	Antibiotic
Ceftazidime	1.35	194.1	545.6	8.68	-9.68	-1	Antibiotic
Resazurin	1.79	75.3	229.2	2.80	-2.81	0	Intracellular conversion into a fluorescent product
Ala-NAP	1.88	56.7	215.3	4.02	-3.02	1	Intracellular conversion into a fluorescent product
Cefaloridine	1.96	93.4	415.5	6.14	-6.14	0	Antibiotic
Moxifloxacin	2.01	89.5	401.4	5.87	-5.87	0	Antibiotic
Thiolactomycin	2.41	34.1	210.3	2.39	-2.39	0	Antibiotic
Cloxacillin	2.67	115.6	434.9	4.96	-5.96	-1	Antibiotic
Proflavin	2.80	66.2	210.3	4.03	-3.03	1	Fluorescent probe
Pyronin Y	2.98	15.5	267.4	3.14	-2.14	1	Fluorescent Probe, quenched intracellular fluorescence
Nitrocefin	3.02	181.2	515.5	7.20	-8.20	-1	Antibiotic
Clarithromycin	3.12	184.1	749.0	9.61	-8.61	1	Antibiotic
Acriflavine	3.19	55.9	224.3	4.06	-3.06	1	Fluorescent Probe, quenched intracellular fluorescence
Leu-NAP	3.29	56.7	257.4	4.02	-3.02	1	Intracellular conversion into a fluorescent product
Novobiocin	3.30	198.9	611.6	8.03	-9.04	-1	Antibiotic
ANS	3.35	63.2	298.3	3.71	-4.71	-1	Fluorescent Probe, enhanced intracellular fluorescence
Nile red	3.71	41.9	318.4	3.38	-3.38	0	Fluorescent Probe, enhanced intracellular fluorescence
Telithromycin	3.84	173.1	813.0	9.91	-8.91	1	Antibiotic
Fluorescein	4.13	76.0	332.3	4.16	-4.16	0	Fluorescent probe
DASPEI	4.38	7.12	253.4	3.45	-2.45	1	Fluorescent Probe, enhanced intracellular fluorescence
Hoechst H33342	4.47	74.3	453.6	6.09	-5.09	1	Fluorescent Probe, enhanced intracellular fluorescence
NPN	4.51	12.0	219.3	2.40	-2.40	0	Fluorescent Probe, enhanced intracellular fluorescence
Rifampicin	4.56	221.4	824.0	11.1	-10.1	1	Antibiotic
Ethidium	5.20	55.9	314.4	4.66	-3.66	1	Fluorescent Probe, enhanced intracellular fluorescence
TMA-DPH	5.39	0.0	290.4	4.18	-3.18	1	Fluorescent Probe, enhanced intracellular fluorescence
Rhodamine 6G	5.76	59.9	442.6	4.23	-4.24	0	Fluorescent probe
1,2′-dinaphthylamine	5.77	12.0	269.3	2.70	-2.70	0	Fluorescent Probe, enhanced intracellular fluorescence
DiOC_2_(3)	6.06	29.5	333.4	3.79	-2.79	1	Fluorescent Probe, enhanced intracellular fluorescence
**Other**				
Imipenem	-0.69	118.3	299.4	5.50	-5.50	0	Antibiotic, not a substrate
Resorufin	1.49	58.9	213.2	2.75	-2.75	0	Fluorescent probe
Optochin	3.75	46.8	341.5	4.36	-3.37	1	Quinolone

*Descriptors were calculated for pH 7 using Molecular Operating Environment (MOE), 2013.08; Chemical Computing Group Inc., 1010 Sherbooke St. West, Suite #910, Montreal, QC, Canada, H3A 2R7, 2014*.

*^a^Logarithm of partition coefficient between *n* -octanol and water (o/w); ^b^Total Polar Surface Area; ^c^Molecular Weight; ^d^Partial positive charge; ^e^Partial negagtive Charge; ^f^net Partial Charge*.

*^g^Examples for which eﬄux inhibition was shown are listed. Other substrates may act as inhibitors too*.

The expression of RND pumps is regulated as a response to external stress factors such as reactive oxygen species (MexAB-OprM, MexXY-OprM), reactive nitrogen species (MexEF-OprN), and other agents imposing stress to the bacterial cell like membrane damaging agents (MexCD-OprJ) or ribosome blocking substances (MexXY-OprM), (Grkovic et al., 2002; Lister et al., 2009; Morita et al., 2014; Poole, 2014). Thus, eﬄux pumps may be part of a versatile protection mechanism against cellular stress that works not only in response to naturally occurring signals but also against antibiotics.

Increased pump expression can be linked to decreased porin expression as for example in the *nfxC* mutants of *P. aeruginosa*. In these mutants OprD expression is downregulated, which impairs carbapenem uptake and MexEF-OprN expression is upregulated, which affects fluoroquinolone export (Fukuda et al., 1990, 1995; Davin-Regli et al., 2008; Nishino et al., 2009; Castanheira et al., 2014).

One possibility to circumvent eﬄux is the development of antibiotics that are not pump substrates, or are only poorly affected by pump activity (e.g., Hayashi et al., 2014). Alternatively, one may look for molecules that inhibit pumps and can be used as adjuvants in combination with antibiotics (e.g., Olivares et al., 2013). For both strategies it is crucial to understand the molecular properties that define pump substrates. This review describes experimental procedures that are sensitive to RND eﬄux pump activity and allow conclusions on substrate recognition by RND eﬄux pumps. The described methods include specifically designed eﬄux assays but also assays which were developed for other purposes than eﬄux studies but revealed substrates of eﬄux pumps (e.g., probes for membrane integrity have been found to be eﬄux pump substrates). Substrate recognition by MexB from *P. aeruginosa* can often not (or not yet) be investigated with the methods described for AcrB in *E. coli* but can be modeled with *in silico* methods based on experimental data. Specific data about substrate recognition by MexB from *P. aeruginosa* are still limited. Therefore we discuss in detail results obtained also for AcrB when they describe similarities between the two RND transporters. It is paradigmatic that to date and to our knowledge there is only a single computational study addressing the molecular aspects of MexB-substrate interactions.

## Impact of Eﬄux on Antibiotic Activity

A direct impact of eﬄux on antibiotic activity on *P. aeruginosa* was shown for a core set of RND pumps by eﬄux-pump deletion mutants and could be confirmed by mutants that overexpress selected RND systems (Li et al., 1995; Lomovskaya et al., 1999; Masuda et al., 2000a; Poole, 2000, 2004, 2005, 2013; Schweizer, 2003). Susceptibility of *P. aeruginosa* towards many antibiotics has been restored when the four systems that are most relevant for antibiotic resistance (MexAB-OprM, MexCD-OprJ, MexEF-OprN, and MexXY-OprM) have been deleted (Morita et al., 2001; Kumar et al., 2006). These *P. aeruginosa* RND pumps have overlapping but not identical substrate ranges as mentioned in the Section *Introduction*. RND eﬄux pumps have individual substrate specificities that include amphiphilic molecules (e.g., MexB) but also hydrophobic solutes (e.g., MexB, MexD) and the hydrophilic polycationic aminoglycosides (MexY), described to enter cells by a self-promoted mechanism (Hancock, 1997). Note that most antibiotics are amphiphilic compounds with hydrophobic parts that are required to partition into a membrane but that there are considerable differences with regard to the impact of eﬄux on activity even within classes of antibiotics (Hancock, 1997; Nikaido, 2003; Delcour, 2009; Brown et al., 2014).

An effort to make tetracyclines that are not substrates of tetracycline-specific pumps of the major facilitator superfamily (MFS), led to the development of tigecycline (Chopra, 2002). However, tigecycline was still a substrate of the RND pumps MexY, MexB, and MexD in *P. aeruginosa* (Dean et al., 2003; Visalli et al., 2003; Chollet et al., 2004). This observation illustrates the flexibility of RND eﬄux pumps but it does not mean that a given RND transporter would accept all antibiotics of the same class. The macrolides erythromycin and clarithromycin have been found to be better substrates of AcrB of *E. coli* and of *Enterobacter aerogenes* than the ketolide telithromycin (Dean et al., 2003; Visalli et al., 2003; Chollet et al., 2004). RND pumps in *P. aeruginosa* have been shown by *in vitro* susceptibility studies to transport substrates of the quinolone family with differential preference (Li et al., 1995; Kohler et al., 1997; Lomovskaya et al., 1999; Masuda et al., 2000a; Griffith et al., 2006; Dunham et al., 2010; Morita et al., 2015). Fluoroquinolones like levofloxacin, ciprofloxacin, norfloxacin, and others, have been reported to be most efficiently exported by MexEF-OprN and MexCD-OprJ and with less efficiency by MexAB-OprM and MexXY-OprM (or OprA). Non-fluorinated quinolones (e.g., nalidixic acid, piromidic acid, pipemidic acid, cinoxacin, oxolinic acid, and flumequine) have been shown to be preferentially transported by MexEF-OprN, less efficiently by MexCD-OprJ and MexAB-OprM, and least efficiently by MexXY-OprM (Kohler et al., 1997; Masuda et al., 2000b). It has been concluded from these results that fluoroquinolones with a positive charge and an electronegative fluorine atom are predominantly recognized by MexCD-OprJ, whereas quinolones without the fluorine atom are preferred substrates of MexEF-OprN. This result was in agreement with earlier work, suggesting that MexCD-OprJ expels amphiphilic substrates with a positive charge that can partition into a membrane (Masuda et al., 1996; Nikaido, 1996).

Studies on β-lactam eﬄux by AcrB in *Salmonella enterica* serovar Typhimurium have indicated that RND eﬄux pumps somehow recognize the hydrophobicity of a substrate (Nikaido et al., 1998; Ferreira and Kiralj, 2004). The studies showed that eﬄux efficiency varied with the lipophilicity of the β-lactam side chain and that molecules with more hydrophobic side chains were preferred substrates. Further work with β-lactams shed light on substrate specificity among RND pumps in *P. aeruginosa*. MexB transported a broad spectrum of β-lactams including penicillins, cephems, and carbapenems but not imipenem (Masuda et al., 2000b, 2001; Baum et al., 2009). MexD had a slightly narrower spectrum, which excluded the MexB substrates carbenicillin, sulbenicillin, ceftazidime, and moxalactam (Li et al., 1994; Masuda et al., 2000b). MexY had a yet narrower spectrum, which further excluded cefsulodin, flomoxef, and aztreonam (Masuda et al., 2000b; Hocquet et al., 2006). As carbenicillin and sulbenicillin have a negative charge that the other β-lactams in theses studies do not have, it seems that MexB accepts substrates with negative charges that MexD and MexY do not recognize. The finding that certain variants of MexD had altered substrate specificity has suggested that the recognition likely takes place via electrostatic interactions. MexD Q45K and MexD E89K with additional positively charged lysine residues have conferred resistance to the negatively charged carbenicillin, aztreonam, and ceftazidime (Mao et al., 2002). A role of charged residues in substrate recognition has been shown for AcrD too based on the fact that the binding pocket of AcrD contains an arginine residue (R625) not found in the mostly hydrophobic pocket of AcrB. Resistance to negatively charged β-lactams increased significantly when an arginine residue was introduced at the corresponding place in AcrB (I626R). Removal of a glutamic acid residue from the AcrB pocket (E673G), a residue not present at this position in AcrD, further enhanced the protective effect and helped to explain the specificity of AcrD for small hydrophilic substrates such as the anionic β-lactams carbenicillin, aztreonam, and sulbenicillin (Wehmeier et al., 2009; Kobayashi et al., 2014).

**FIGURE 1:**
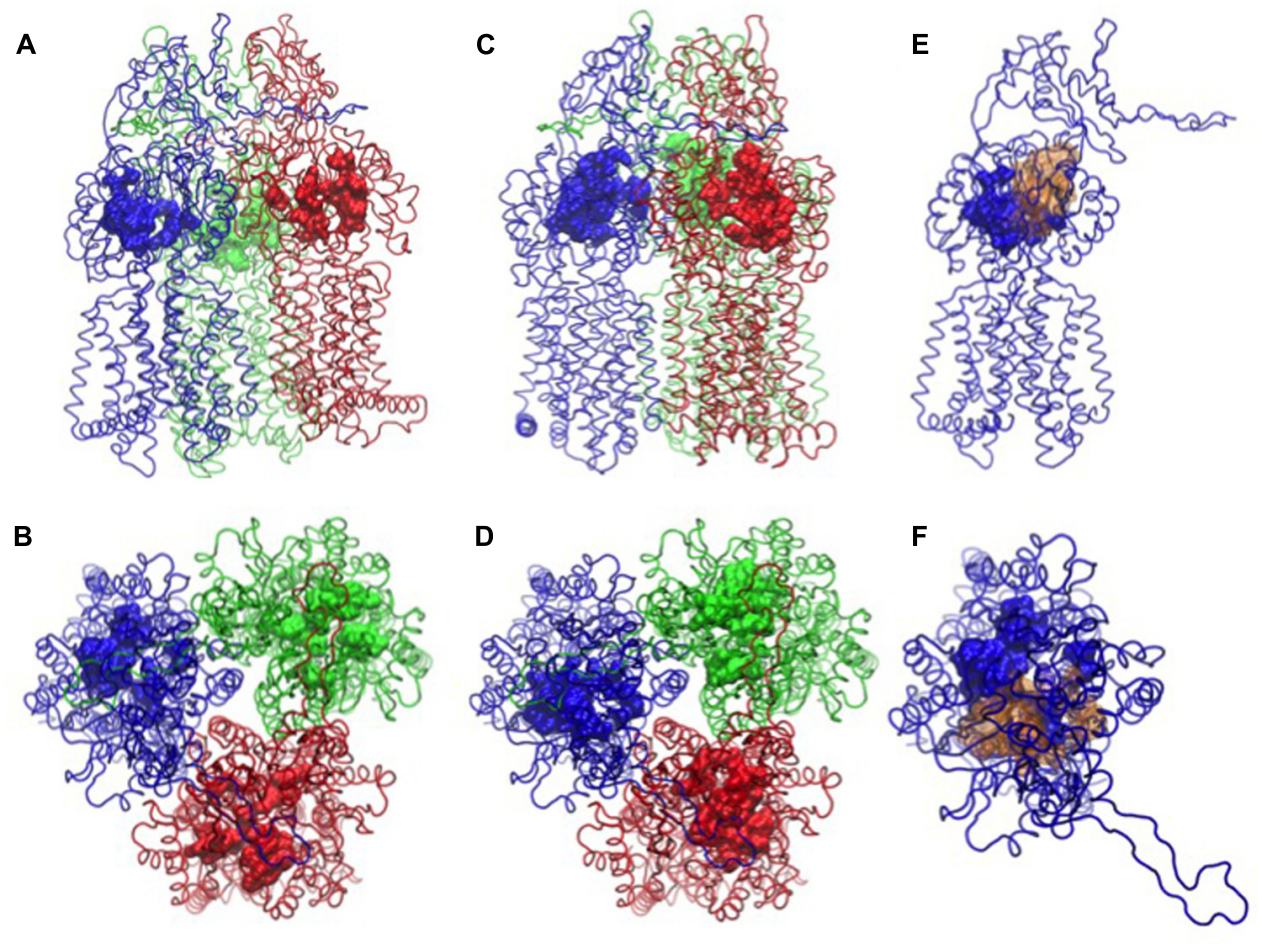
**Binding pockets of MexB [PDB code: 2V50 (Sennhauser et al., 2009)]**. The monomers L, T, and O are in tube representation and colored in blue, red, and green, respectively. In **(A,B)** side and top views of MexB with the distal binding pocket depicted as surface, in **(C,D)** the access binding pocket is highlighted as surface. In **(E,F)** the side and top views of L monomer are shown with access and distal binding pocket colored as blue and yellow surfaces, respectively. All the atomic-level figures were rendered using Visual Molecular Dynamics (VMD; Humphrey et al., 1996).

Carbapenem activity was not uniformly affected by eﬄux pumps either. Meropenem was sensitive to overexpression of MexAB-OprM, MexCD-OprJ, or MexXY-OprM, whereas imipenem was not significantly affected (Masuda et al., 2000b; Ong et al., 2007; Riera et al., 2011; Castanheira et al., 2014). Eﬄux-pump expression levels in clinical isolates of *P. aeruginosa* indicated that MexAB-OprM and MexXY-OprM were most relevant for meropenem activity, followed by MexEF-OprN and with the lowest impact by MexCD-OprJ (Pai et al., 2001; Giske et al., 2005; Riera et al., 2011). A computer simulation of imipenem and meropenem transport by MexB suggested that the hydrophobicity and the flexibility of the side chains were probably responsible for eﬄux sensitivity (Collu et al., 2012b). The study indicated that the more rigid and hydrophobic tail of meropenem made strong interactions with the hydrophobic lining of the distal binding pocket in MexB (see **Figure 1** for the structure of the transporter with two affinity sites) whereas the flexible and more hydrophilic tail of imipenem prevented this interaction (a detailed description of the extrusion pathway is given in the Section *Computational Study*). The computational results have further suggested that imipenem had no pronounced affinity for any particular site in MexB.

The anti-MRSA cephalosporin ceftobiprole is another β-lactam that is poorly recognized by MexB, MexD, and MexF in *P. aeruginosa* (Baum et al., 2009). Overexpression of these eﬄux pumps had only the marginal effect on the activity of ceftobiprole of a twofold increase of the minimum inhibitory concentration (MIC). Overexpression of MexXY caused a fourfold to eightfold increase of the MIC. The data have indicated that ceftobiprole was more efficiently transported by MexY than by MexB (Baum et al., 2009).

It should be pointed out that not all eﬄux substrates can be reliably detected by MIC. MIC may be a misleading indicator for eﬄux for any antibacterial that is effective at a concentration below the concentration required to reach maximal eﬄux velocity *V*_max_. For example, AcrB had only a minor effect on the activity of cefaloridine (Nikaido et al., 1998; Mazzariol et al., 2000). Cefaloridine was later shown to be strongly eﬄuxed by AcrB only at concentrations higher than those required to kill bacteria (Nagano and Nikaido, 2009). The discrepancy was explained by the finding that cefaloridine was eﬄuxed with a high *V*_max_ but also with a strong positive cooperativity. The cefaloridine concentration required to reach half-maximal velocity (288 μM) was much higher than the effective antibacterial dose (Nikaido and Normark, 1987; Nagano and Nikaido, 2009). Note that cefaloridine may not be an exception because other drugs (cephalotin, cefamandole) were also transported in a cooperative manner (Nagano and Nikaido, 2009). The situation may be different in *P. aeruginosa* because its OM has a lower permeability for β-lactams than the OM of *E. coli* (as explained before). This lower permeability leads to higher MIC and to a stronger impact of eﬄux than in *E. coli* and thus, less discrepancy between eﬄux studies and MIC data may be expected.

MIC tests in the presence of the model eﬄux-pump inhibitor (EPI) phenylalanine-arginine β-naphthylamide (PAβN) are often done as a quick check for eﬄux effects (e.g., Mesaros et al., 2007; Pages et al., 2009; Castanheira et al., 2014). Increased antimicrobial activity (i.e., lower MIC) of an antibiotic in the presence of PAβN is taken as an indication that the test compound is an eﬄux substrate. However, detailed studies with PAβN nicely illustrated the complexity of effects that can influence whole cell assays such as MIC determinations. It has already been mentioned in the original publication of PAβN that this agent does not only inhibit eﬄux pumps but also permeabilizes the OM of Gram-negative bacteria (Lomovskaya et al., 2001). This permeabilization property has been confirmed later in separate studies (Iino et al., 2012b; Lamers et al., 2013). Permeabilization of the OM may be mistaken as pump inhibition because facilitated entry of test compounds leads to increased intracellular levels. Pump inhibition and membrane permeabilization by PAβN have been shown to be dose-dependent, separable activities in *E. coli* because AcrB and AcrF have been specifically inhibited at low doses of PAβN, whereas membrane destabilization has been observed at higher concentrations of PAβN (Misra et al., 2015).

The protonophore carbonyl cyanide m-chlorophenylhydrazone (CCCP) is often used in a similar way as PAβN to probe for eﬄux phenomena. RND pumps belong to the class of secondary transporters, which are driven by a transmembrane electrochemical potential of protons (reviewed in Borges-Walmsley et al., 2003; Poole, 2005; Dreier, 2007). CCCP indirectly inhibits secondary eﬄux pumps by dissipating the proton gradient across the inner membrane (Rosen and Kashket, 1978; Levy, 1992). It should be clear that whole cell assays require thorough controls to avoid confusion of pump inhibition with other effects. Susceptibility tests are useful to investigate general substrate recognition patterns whereas elucidation of detailed eﬄux mechanisms requires specifically designed assays.

## Eﬄux Assays

Transport studies generally require at least two compartments and a method to measure concentration changes of a tracer substance. Entire RND eﬄux systems require three compartments (cytosol, periplasm, and cell exterior), which makes whole bacteria an obvious choice for eﬄux assays. In principle, cells can be incubated with any pump substrate and eﬄux can be measured by following the change of substrate concentration inside or outside of the bacteria [e.g., (Kohler et al., 1997; Pumbwe and Piddock, 2000)]. Such a generic method may require the separation of intracellular from extracellular substrate, usually achieved by filtration or centrifugation. Cell lysis may be necessary too before a versatile detection method like liquid chromatography–mass spectrometry (LC–MS) or high performance liquid chromatography (HPLC) can be applied (e.g., Schumacher et al., 2007; Cai et al., 2009). Homogeneous assays use methods that allow substrate quantification without a separation step, which simplifies applications like time-course transport studies or high-throughput tests. Fluorescence was selected as the method of choice to assess fluoroquinolone accumulation in Gram-negative bacteria based on a comparison of various methods (Mortimer and Piddock, 1991). The study showed that steady-state concentrations and times to reach the steady-state condition varied in a substrate-specific manner within the quinolone family.

**FIGURE 2:**
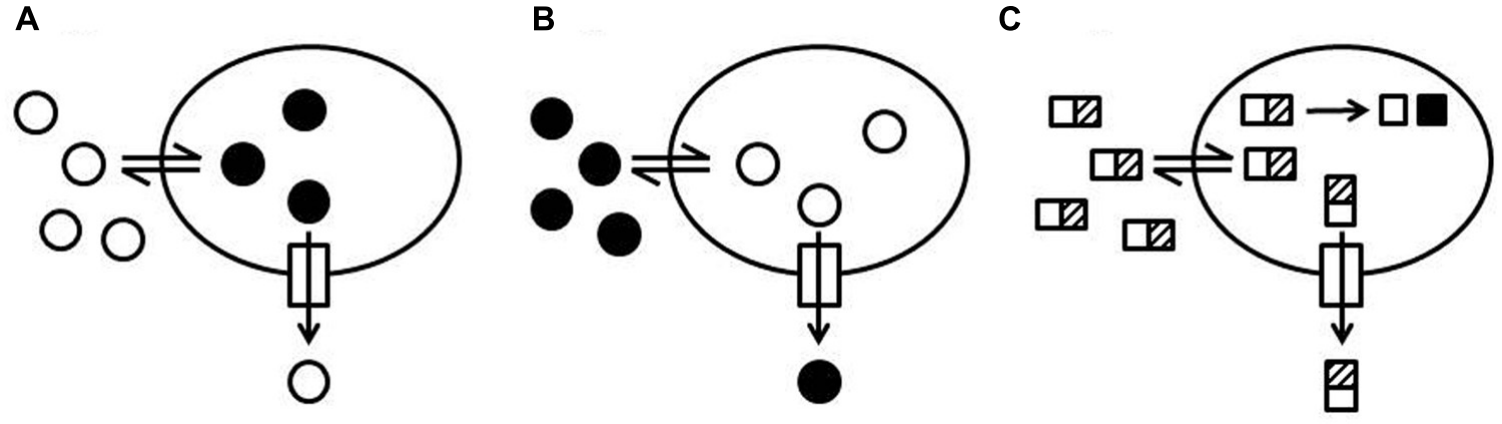
**Schematic representation of eﬄux assay formats. (A)** Increase of fluorescence intensity upon internalization of a probe. A probe with low fluorescence intensity in the medium surrounding the cells (open circles) is taken up by cells (double arrow). The fluorescence intensity of the probe increases when the probe is internalized (black circles) and interacts with cellular structures such as DNA, proteins, or membranes. Eﬄux of the probe (arrow through the box to the outside) causes a decrease of the total fluorescence and eﬄux inhibition causes an increase of the total fluorescence. **(B)** Quenching of fluorescence intensity upon internalization of a probe. A fluorescent probe (black circles) is added to the medium surrounding the cells. The fluorescence intensity of the probe decreases when the probe is taken up by the cells (open circles) and interacts with cellular structures (e.g., DNA and RNA). Eﬄux of the probe causes an increase of the total fluorescence and eﬄux inhibition causes a decrease of the total fluorescence. **(C)** Intracellular conversion of a probe into a fluorescent product. A non-fluorescent probe (rectangles with a hatched box) is added to the medium surrounding the cells. The probe can penetrate into the cells where it is enzymatically converted (e.g., cleaved or reduced) into a fluorescent product (black squares). The conversion rate of the intact probe into the fluorescent product depends on the intracellular concentration of the intact probe. Eﬄux of the intact probe slows down the production of the fluorescent product and eﬄux inhibition increases the rate of fluorescent product generation.

Many protocols use molecules whose fluorescent properties are sensitive to their environment and change upon entry into a cell (**Figures 2A,B**). A prominent example is ethidium bromide (EtBr) since the quantum yield of the fluorescence increases when ethidium intercalates into DNA (LePecq and Paoletti, 1967; Mine et al., 1999; Morita et al., 2001; Li et al., 2003). Active eﬄux causes reduced intracellular levels of EtBr and as a consequence a decrease of fluorescence whereas inhibition of eﬄux pumps is recorded as a signal increase (**Figure 2A**). The same assay principle (**Figure 2A**) applies to 1-anilinonaphtalene-8-sulphonate (ANS) accumulation because the fluorescence quantum yield increases when ANS binds to hydrophobic structures (e.g., proteins, membranes) in the cell. ANS is a substrate of MexD and has been used to study this transporter in *P. aeruginosa* (Walmsley et al., 1994; Mao et al., 2002; Kamal et al., 2013). Another example is 1,2′-dinaphthylamine which has been used to measure AcrB eﬄux in *E. coli* because it is a substrate of AcrB and becomes strongly fluorescent when it partitions into a phospholipid bilayer (Bohnert et al., 2011a).

A widely used fluorophore with increased intracellular fluorescence is the bis-benzamide dye Hoechst H33342 which becomes more fluorescent upon binding to DNA (Loontiens et al., 1990). H33342 was successfully used for accumulation studies with several Gram-positive bacteria, Enterobacteriaceae, and *Acinetobacter baumannii* (van den Berg van Saparoea et al., 2005; Garvey and Piddock, 2008; Richmond et al., 2013). Transport of H33342 by intact *P. aeruginosa* has been difficult to measure because of the low OM permeability for such hydrophobic dyes (Loh et al., 1984; Ocaktan et al., 1997; Germ et al., 1999). However, MexB readily transported H33342 when it was expressed in an *E. coli* host (Welch et al., 2010; Ohene-Agyei et al., 2012). This example shows that whole cell assays reflect a sum of events, which often need to be dissected to identify unambiguously substrates of a given eﬄux pump.

Another group of eﬄux probes allows eﬄux measurements based on a signal decrease upon intracellular accumulation (**Figure 2B**; e.g., Mao et al., 2002; Nakashima et al., 2013). Examples are the anthracycline cancer drug doxorubicin, whose fluorescence is quenched when the drug is internalized, Pyronin Y, whose intracellular fluorescence is quenched because of binding to RNA (Nishino and Yamaguchi, 2002; Nakashima et al., 2013), and acriflavine, whose fluorescence intensity decreases when it is bound to DNA (Chen et al., 2002). Pyronin Y has been successfully used for transport studies with MexD in *P. aeruginosa* (Mao et al., 2002).

Intracellular conversion of a substrate into a traceable entity is another elegant way to develop homogeneous eﬄux assays (**Figure 2C**). The non-fluorescent, blue-colored resazurin (sold under the trade name Alamar Blue^®^) is reduced to the fluorescent, pink-colored resorufin in live cells by oxidoreductases (Gonzalez and Tarloff, 2001; Rampersad, 2012). The conversion is an indicator mainly for the presence of NADH but also for NADPH and FADH and is routinely used to detect living cells. Resazurin can be used to monitor bacterial growth because conversion rates depend on cell density. However, the method does not work equally well with all microorganisms and is not recommended for *P. aeruginosa*. It was shown that resazurin is a substrate of MexB in *P. aeruginosa* and of AcrB in *E. coli* and it was suggested that eﬄux pump activity in *P. aeruginosa* may account for the difficulties encountered with resazurin (Vidal-Aroca et al., 2009). Vidal-Aroca et al. (2009) showed that under conditions where Pseudomonas do not grow (i.e., in phosphate buffered saline), resazurin can be used to measure eﬄux pump activity. The assay was useful to identify mefloquine as an inhibitor of MexB and AcrB and to show that optochin, another quinoline derivative, was not an inhibitor of these pumps.

The non-fluorescent alanine-β-naphthylamide (Ala-NAP) and leucine-β-naphthylamide (Leu-NAP) are enzymatically converted to the highly fluorescent β-naphthylamide by cellular aminopeptidases (Marks et al., 1981; Butler et al., 1993). Assays for RND eﬄux were developed with Ala-NAP and Leu-NAP and used for the development of peptidomimetic EPI including the model EPI PAβN (Lomovskaya et al., 2001; Mao et al., 2002).

Fluorescein-di-β-D-galactopyranoside (FDG) is hydrolyzed in the cytoplasm of *E. coli* by β-galacotsidase to produce fluorescein (Russo-Marie et al., 1993; Fieldler and Hinz, 1994; Yang and Hu, 2004). Both FDG and fluorescein were shown to be eﬄux-pump substrates and could be used to develop transport assays with *E. coli* cells expressing the pseudomonal MexAB-OprM and MexXY-OprM eﬄux systems (Matsumoto et al., 2011). Intracellular conversion of FDG into fluorescein could even be followed in single-cell mode with a microfluidic device using *E. coli* (Matsumoto et al., 2011; Iino et al., 2012b). FDG cannot be hydrolyzed by *P. aeruginosa* but FDG was useful to study MexB and MexY when they were expressed in an *E. coli* host (Iino et al., 2013). Another approach made use of femtoliter droplet arrays to monitor FDG based eﬄux in single *E. coli* cells (Iino et al., 2012a, 2013).

The following paragraphs describe fluorescent dyes, which were used as probes for membrane integrity or membrane potential and were later found to be substrates of eﬄux pumps. The physicochemical properties of these fluorescent dyes provide information on substrate recognition by eﬄux pumps irrespective of the original use of the probe.

The lipophilic membrane partitioning dye *N*-phenyl-1-naphthylamine (NPN) is useful as a probe for membrane integrity because the amount of NPN that can partition into the phospholipid membrane and hence the fluorescence signal increases when the structure of the OM is disturbed (Loh et al., 1984; Vaara, 1992; Lomovskaya et al., 2001). NPN was shown to be a substrate of AcrB in *E. coli* and of MexB in *P. aeruginosa* (Sedgwick and Bragg, 1996; Ocaktan et al., 1997; Lomovskaya et al., 2001; Murakami et al., 2004; Cai et al., 2009).

Fluorescent potentiometric probes such as the anionic oxonols and the cationic carbocyanines and rhodamines are useful to measure changes of bacterial membrane potentials (Shapiro, 2000). The overall fluorescence signal of these so called slow-response dyes depends on the transmembrane distribution, which is sensitive to changes of the electrochemical potential across the membrane. Rhodamine measurements in *P. aeruginosa* were sensitive to the expression of the RND pumps MexAB-OprM, MexCD-OprJ, and MexHI-OpmD (Morita et al., 2001; Sekiya et al., 2003). The physicochemical features of these fluorophores, required to partition into a membrane, are apparently recognized by RND eﬄux-pumps. Indeed, eﬄux assays were reported in various systems with dyes like the uncharged NPN (MexB of *P. aeruginosa*, AcrB of *E. coli*) or the cationic 1-(4-trimethylammoniumphenyl)-6-phenyl-1,3,5-hexatriene (TMA-DPH, MexB of *P. aeruginosa*) and 2-(4-dimethylamino)styryl-*N*-ethylpyridinium iodide (DASPEI, AcrB of *E. coli*, MexB of *P. aeruginosa*), (Sedgwick and Bragg, 1996; Ocaktan et al., 1997; Lomovskaya et al., 2001; Murakami et al., 2004; Cai et al., 2009). The properties of the dyes that have been identified as substrates of MexB are listed in **Table 1**.

Substrate export can be directly followed from pre-loaded cells. Alternatively, pump activity can be deduced from intracellular accumulation of externally added substrates, which requires less preparation but reflects eﬄux in an indirect manner only and includes the hurdle of cellular uptake. Direct eﬄux measurements require pre-loading of cells with substrate. This extra complication pays off because eﬄux-related effects can be distinguished from permeation phenomena. The lipophilic dye Nile Red is basically non-fluorescent in aqueous solutions but becomes fluorescent in intracellular, non-polar environments (Greenspan and Fowler, 1985). Nile Red was used for eﬄux measurements of AcrAB/TolC in *E. coli* (Greenspan and Fowler, 1985; Bohnert et al., 2010, 2011b). When *E. coli* cells were concomitantly loaded with substrate and inhibitor for competition studies, the strongest inhibition was seen with doxorubicin and minocycline, comparable to the effect of PAβN. Many other antibiotics caused slower Nile Red eﬄux, which is indicative of competitive transport. These results provided further evidence for specific substrate-pump interactions and corroborated the notion of substrate-specific inhibitor efficacy. Adaptation of the Nile Red assay for the use with *P. aeruginosa* would most likely not be straight forward because Nile Red was shown to stain extracelluar rhamnolipids made by *P. aeruginosa* (Morris et al., 2011). Strains with deletions of rhamnolipid synthesis genes (rhlAB) may be helpful even if the swarming motility patterns influenced by rhamnolipids would be changed (Deziel et al., 2003; Caiazza et al., 2005). However, pre-loading of *P. aeruginosa* has been achieved with TMA-DPH (Ocaktan et al., 1997). Several other dyes (doxorubicin, rhodamin for YhiUV, NPN, 1,2′-dinaphthylamine and DASPEI for AcrB) have been used to load *E. coli* for eﬄux assays (Lomovskaya et al., 2001; Nishino and Yamaguchi, 2002; Bohnert et al., 2011a).

Quantitative measurement of substrate transport is crucial for the characterization of eﬄux pumps. Kinetic analysis of EtBr transport in *E. coli* has shown that uptake rates increased and eﬄux rates decreased when AcrB was deleted (Xu et al., 2003; Viveiros et al., 2008; Paixao et al., 2009). EtBr transport by MexB has been shown in whole *P. aeruginosa* and an impressive turnover rate of 500 s^-1^ has been determined for MexB in EtBr eﬄux assays when the number of pumps was estimated by immunoblotting methods (Ocaktan et al., 1997; Narita et al., 2003). A link of MexCD-OprJ expression with EtBr eﬄux from *P. aeruginosa* has been demonstrated by Morita et al. (2001, 2003) when they showed that significant EtBr eﬄux was only measurable after induction of MexCD-OprJ. MexCD-OprJ expression was inducible with tetraphenyl phosphonium, EtBr, rhodamine 6G, acriflavine, benzalkonium chloride, and chlorhexidine gluconate but not with various antibiotics including substrates of MexD (i.e., norfloxacin, tetracycline, chloramphenicol, streptomycin, or erythromycin).

A thorough quantitative analysis of eﬄux was achieved with β-lactams and intact *E. coli* cells (Nagano and Nikaido, 2009). The method relies on the hydrolysis of β-lactams by periplasmatic β-lactamases and would probably not be directly applicable to *P. aeruginosa* because of the about 10–100 times lower permeability of the OM for β-lactams (Angus et al., 1982; Yoshimura and Nikaido, 1982; Parr et al., 1987). Under these circumstances it would be very difficult to saturate the eﬄux pumps and the method may be restricted to a few suitable β-lactams. Hydrolysis rates of β-lactams were measured in intact cells to calculate the periplasmic antibiotic concentration based on known kinetic parameters of β-lactamases. Maximal velocity and dose–response curves depended on the nature of the substrate. Positive cooperativity was detected with cefamandole, cephalotin, and cefaloridin but not with nitrocefin (Nagano and Nikaido, 2009). Cooperativity agrees with the current model of the functional complex of AcrAB-TolC where multiple substrate binding sites reside within three AcrB monomers that adopt different structural conformations in a concerted way during substrate transport (Seeger et al., 2006; Murakami et al., 2006; Sennhauser et al., 2007). Nitrocefin transport was the slowest in the series, which could be explained by the rather strong binding of nitrocefin with its two aromatic rings to AcrB (*K*_m_ of 5 μM). No significant eﬄux could be measured with cefazolin, which has two hydrophilic substituents. Previous work has shown that MIC of cloxacilin, which has a lipophilic side, chain dropped from 256 to 2 mg/L in *S. enterica* serovar Typhimurium and from 512 to 2 mg/L in *E. coli* upon inactivation of AcrB whereas no change was observed for the MIC of the hydrophilic cefazolin (Nikaido et al., 1998; Mazzariol et al., 2000). MIC values and eﬄux studies strongly suggested that AcrB from *E. coli* and *S. enterica* serovar Typhimurium have a preference for hydrophobic β-lactams.

Specific mode-of-action studies ask for less complex assay systems than entire bacterial cells. The activity of different bacterial membrane transporters could be studied with membrane vesicles. Vesicles proved to be very valuable tools for the study of tetracycline-specific pumps (e.g., Yamaguchi et al., 1990). Tetracycline-specific pumps belong to the MFS class of transporters spanning a single cell membrane (Chopra and Roberts, 2001), whereas RND pumps are multi-subunit complexes made of pump subunits (e.g., MexB), an OM channel (e.g., OprM), and an adaptor subunit (e.g., MexA). The pump is formed by three monomers placed in the inner membrane and protrudes into the periplasm (Murakami et al., 2002, 2006; Seeger et al., 2006; Sennhauser et al., 2007, 2009). The OM channel, formed by three monomers, passes the OM linking the periplasmic space with the outside of the cell (Koronakis et al., 2000; Akama et al., 2004a; Phan et al., 2010). The third subunit is an adaptor protein that is required for the assembly of the functional complex which spans the inner membrane, the periplasmic space, and the OM. The stoichiometry of the subunits (MexB:MexA:OprM or AcrB:AcrA:TolC) is currently discussed with evidence for 3:6:3 (Du et al., 2014, 2015; Kim et al., 2015) or for 3:3:3 (Symmons et al., 2009; Trepout et al., 2010). The models are based on electron microscopy data at resolutions around 20 Å and on docking calculations. A higher-resolution crystal structure of the assembled eﬄux systems will likely provide a clear-cut response to the controversy regarding the stoichiometry and the construction of the tripartite complex.

The isolated pump subunits AcrB and AcrD from *E. coli* and MexB from *P. aeruginosa* were reconstituted in vesicles (Zgurskaya and Nikaido, 1999; Aires and Nikaido, 2005; Verchère et al., 2014, 2015) but reconstitution of a functional tripartite RND pump complex for *in vitro* transport studies is a truly difficult task that has been achieved only recently (Ntsogo-Enguene et al., 2015; Verchère et al., 2015). Competition studies with AcrB-containing vesicles have indicated that the bile salts taurocholate and glycocholate inhibited transport of a fluorescent phospholipid more efficiently than erythromycin or cloxacillin did (Zgurskaya and Nikaido, 1999). Chloramphenicol was identified in the same study as a substrate that did not inhibit phospholipid eﬄux. These results suggested that known AcrB substrates were transported with different efficiency reflecting specific substrate recognition patterns. Functional MexAB-OprM complexes have been assembled *in vitro* in a liposome system combining proteoliposomes with OprM and proteoliposomes with MexAB in a way that MexAB-OprM was able to transport EtBr driven by a proton gradient (Ntsogo-Enguene et al., 2015). A related approach used vesicles with MexB and bacteriorhodopsin, a light activated proton pump from *Halobacterium salinarium* (Verchère et al., 2014). Light activation caused bacteriorhodopsin to build up a proton gradient as the energy source for MexB. A change of pH was then taken as indication for substrate transport. The system confirmed that H33342 is a substrate of MexB and suggested that MexA was required for proton-coupled transport.

## Eﬄux Inhibitors

Many chemically diverse EPI have been reported and their selectivity for pumps can provide insight into specific inhibitor–pump interactions (reviewed in Zechini and Versace, 2009; Van Bambeke et al., 2010). Inhibitors have been derived from natural products, antibiotics, drugs originating from other therapeutic areas or have been newly developed. PAβN and more advanced peptidomimetics of the same series are among the best studied examples (Lomovskaya et al., 2001; Mao et al., 2001; Renau and Lemoine, 2001; Renau et al., 2002; Watkins et al., 2003; Kriengkauykiat et al., 2005). Selectivity for pumps indicated specific inhibitor–pump interactions. For example, PAβN was broadly active against MexAB-OprM, MexEF-OprN, MexCD-OprJ, and MexXY-OprM in *P. aeruginosa* as well as against AcrAB-TolC in several species of the Enterobacteriaceae family while the pyridopyrimidine eﬄux inhibitor D13-9001 had a much narrower spectrum with specificity for AcrB and MexB (Nakashima et al., 2013). PAβN has been shown to have synergistic activity with levofloxacin against wild-type *P. aeruginosa* or against *P. aeruginosa* strains overexpressing any of MexAB-OprM, MexCD-OprJ, MexEF-OprM, or MexXY (Lomovskaya et al., 2001). On the other hand, PAβN had no significant effect on carbenicillin or EtBr, which are substrates of MexB too, suggesting multiple binding sites for different substrates. An antagonistic effect of PAβN with aminoglycosides has been observed in the presence of MexXY-OprM, which was explained by the induction of this eﬄux pump by PAβN (Mao et al., 2001). PAβN was shown to be an eﬄux substrate likely to act by competition with other substrates for transport (Pages et al., 2005; Lomovskaya and Bostian, 2006; Mahamoud et al., 2007). D13-9001 was hardly transported but was shown to inhibit eﬄux by high affinity binding to a specific site (Nakashima et al., 2013). Many substances cannot be unambiguously classified as substrates or as inhibitors because there seems to be a gradual transition of properties and many molecules have both characteristics. Minocycline is a substrate of several RND pumps such as AcrB from *E. coli*, *Proteus mirabilis*, *Morganella morganii*, MexB, MexD, and MexY in *P. aeruginosa*, or AdeB and AdeJ in *A. baumannii* (Dean et al., 2003; Visalli et al., 2003; Hirata et al., 2004; Ruzin et al., 2005; Damier-Piolle et al., 2008) but it also acted as an inhibitor of nitrocefin eﬄux by AcrB (Takatsuka et al., 2010). Trimethoprim, an antibiotic that has been shown to be transported by several RND eﬄux pumps (i.e., MexB, MexD, and MexF in *P. aeruginosa*, AdeB, AdeJ, AdeG in *A. baumannii*, and BpeF in *Burckholderia pseudomallei*), inhibited the eﬄux of H33342 by AcrB in *S. enterica* serovar Typhimurium (Kohler et al., 1996; Maseda et al., 2000; Magnet et al., 2001; Coyne et al., 2010; Piddock et al., 2010; Amin et al., 2013; Podnecky et al., 2013). In fact, antibiotics could be modified to become eﬄux inhibitors as described in the case of fluoroquinolones, tetracyclines, or aminoglycosides (reviewed in Van Bambeke et al., 2010). In general, EPI have aromatic moieties and contain ionizable groups, which are structural features that are reminiscent of substrate characteristics deduced from antibiotic susceptibility tests (Poole, 2004, 2005).

Transport measurements are useful to identify molecules that interact with eﬄux pumps but investigation of interactions at a molecular level and precise mode-of-action studies require more refined methods.

## Binding Studies and Crystal Structures

Binding of fluorescent substrates to purified AcrB in detergent solution could be measured by fluorescence polarization (Su and Yu, 2007). AcrB bound rhodamine 6G, ethidium, and proflavin, with similar strength (*K*_D_ of 5.5, 8.7, and 14.5 μM, respectively). Binding of ciprofloxacin was significantly weaker with a *K*_D_ of 74.1 μM. Competition studies have indicated that different binding sites may exist for different antibiotics. Experiments with purified AcrB which was immobilized to a surface, yielded *K*_D_ values of 530 μM for novobiocin and of 110 μM for the EPI MC-207,110 (PAβN; Tikhonova et al., 2011). Binding studies confirmed specific interaction of substrates or inhibitors with the pump subunit and revealed substrate-specific variation of binding strength (Vargiu and Nikaido, 2012; Vargiu et al., 2014).

Many molecules have been described to be eﬄux substrates or inhibitors but only a small subset thereof could be crystallized in complex with RND pumps (Ruggerone et al., 2013a). Nevertheless, X-ray structures have provided detailed information on substrate-pump interactions (Murakami et al., 2006; Seeger et al., 2006; Sennhauser et al., 2007, 2009; Nakashima et al., 2011; Eicher et al., 2012). A detailed description of the structures is provided in the next section where computational simulation approaches are discussed. The porter domain of the inner membrane transporter protrudes into the periplasmic space (**Figure 1**) and mediates substrate specificity as predicted from genetic studies with AcrB, AcrD, MexB, MexD and MexY (Nikaido, 2011). Gene segments coding for the periplasmatic loops of the pump subunit were swapped between AcrB and AcrD of *E. coli*, between MexB and MexY of *P. aeruginosa*, and between AcrB of *E. coli* and MexB of *P. aeruginosa* (Elkins and Nikaido, 2002; Tikhonova et al., 2002; Eda et al., 2003). Altered substrate specificities of the new constructs suggested that substrate recognition was predominantly determined by the periplasmic region of the pump. Site directed mutagenesis in *P. aeruginosa* confirmed substrate recognition sites in the periplasmatic loops of MexB and MexD (Mao et al., 2002; Middlemiss and Poole, 2004; Wehmeier et al., 2009). Q34K, E89K, and N67K mutations in MexD have led to the transport of negatively charged β-lactams that are not recognized by the wild type transporter (as discussed in the Section *Impact of Eﬄux on Antibiotic Activity*; Mao et al., 2002). A direct substrate contact for macrolide recognition was proposed for an asparagine residue in a phenylalanine-rich distal binding pocket in MexB (Wehmeier et al., 2009).

The high molecular weight substrates rifampicin, erythromycin, and doxorubicin dimers bound to an access (or proximal) binding pocket (AP) shown in **Figures 1A,B**, which is located close to the protein/periplasm interface (Nakashima et al., 2011; Eicher et al., 2012). The low molecular weight substrates minocycline, dodecyl-α-D-maltoside and doxorubicin have been found in a distal (or deep) binding pocket (DP) displayed in **Figures 1C,D**. AP and DP have also been proposed as two successive locations visited by a substrate during the translocation cycle. The two pockets are separated by a flexible loop (G-loop or switch loop) with two phenylalanine residues (Nakashima et al., 2011; Eicher et al., 2012). Substrate recognition is governed by a phenylalanine-rich hydrophobic region in the distal pocket. Minocycline, rifampicin, and erythromycin, made direct contact with phenylalanine residues (Murakami et al., 2006; Nakashima et al., 2011; Eicher et al., 2012). The MexB-specific pyridopyrimidine eﬄux inhibitor D13-9001 bound to a narrow pit in the phenylalanine cluster of the distal pocket making π-π-stacking interactions (Nakashima et al., 2013). The hydrophilic part of the inhibitor interacted with hydrophilic and ionic residues close to the binding site of minocycline and doxorubicin (Nakashima et al., 2013). Binding to MexY was sterically hindered by a tryptophan in agreement with the phenotypic specificity for MexB over MexY (Nakayama et al., 2003; Yoshida et al., 2007). The binding pocket of AcrD contains several oxygens in contrast to the mostly hydrophobic pocket of AcrB, which could explain the specificity of AcrD for small basic, hydrophilic substrates such as the anionic β-lactams carbenicillin, aztreonam, and sulbenicillin (Kobayashi et al., 2014). Domain swapping experiments have shown that the specificity of MexB for anionic β-lactams, which are not recognized by MexY, is determined by the periplasmatic domain of the pump subunit (Eda et al., 2003). Although, the complexity of substrate recognition by RND eﬄux systems has hindered the establishment of a straightforward criterion for the definition of good and poor substrates, it is possible that MexY, as shown for MexD and described before (Mao et al., 2002), does not have the required binding places (i.e., positively charged amino acid residues) in the substrate binding sites to accept negatively charged β-lactams. It cannot be excluded that other, subtle differences in the RND transporter-compound interaction patterns might affect the recognition and the transport at different extrusion stages and positions in the transporters (see **Figure 1** where the different affinity sites are shown). Examples for such subtle but crucial interactions are imipenem and meropenem interacting with MexB (see Section *Computational Study*).

Mutagenesis of D133 in MexY of *P. aeruginosa* (D113A and D133S) has compromised resistance to several aminoglycosides but not to spectinomycin (Lau et al., 2014). The mutation of Y613A in the same protein affected resistance to aminoglycosides but not to erythromycin. This finding supported the view that substrate recognition is governed by binding to specific sites on the extrusion pathway because D133 and Y613 are located in a region of MexY that structurally corresponds to the proximal binding pocket of AcrB.

The experimental data discussed in this review, have provided evidence that hydrophobic moieties are a key property recognized by RND pumps and that hydrophilic parts of a substrate fit into mainly hydrophilic cavities adjacent to a phenylalanine-rich, hydrophobic pocket. Substrates of MexB, which are discussed in this review, are listed in **Table 1**.

## Computational Study

Simulations have reached an impressive maturity as reflected by the increasing number of publications in the field. In particular, a range of simulation techniques have been developed and successfully applied to describe diverse biological systems. Highly precise quantum mechanical techniques (for example see the reviews of Dal Peraro et al., 2007; Zhou et al., 2010; Sgrignani and Magistrato, 2013; Steinbrecher and Elstner, 2013), the classical force-field-based approaches (Kollman et al., 2000), hybrid methods combining appropriate quantum mechanical and classical descriptions (Senn and Thiel, 2009; Steinbrecher et al., 2012) and multi-scale and statistical mechanical methodologies (Kamerlin et al., 2011; Karplus, 2014) are examples of such computational methods.

Although they have been successfully applied to several systems and used to tackle biology-inspired questions, these *in silico* techniques have been barely used to investigate bacterial eﬄux systems, in particular members of the RND family. Probably hampered by the lack of crystal structures of the whole systems (the single components have been crystallized) and by the complexity of the machineries (they are tripartite systems), computational studies have addressed RND eﬄux systems only recently (Collu and Cascella, 2013; Ruggerone et al., 2013a,b). Classical molecular dynamics (MD) simulations represent a particularly promising technique to cast a glimpse on the dynamic behavior of a protein and its immediate micro-environment. MD simulations offer insight into molecular behavior at a temporal and structural accuracy not reached by any other experimental technique today. Continuous improvement of the techniques is pushing the limits of the simulation processes toward longer simulations and thus to a description of increasingly larger biological systems. Extension of the simulation times has improved the quality of the predictions and allowed more robust evaluation of key features such as free energy of binding, interaction patterns, solvent interactions, and interaction lifetimes. Surely, limitations are still present due to the size of many systems and the length of processes of interest, but specific techniques to overcome these drawbacks are under continuous development. As an example of methods used to bridge the time gap between simulated and real processes involving RND eﬄux systems, we can quote targeted MD simulations (Schlitter et al., 1993), which allows induction of conformational changes between two known states, and metadynamics (Laio et al., 2005; Laio and Gervasio, 2008; Biarnés et al., 2011), which is used to simulate rare events and provides free energy profiles associated with possible processes. Advantages and drawbacks of these biased techniques have been extensively discussed in several publications, to which the interested reader is referred (e.g., Chipot, 2008; Laio and Gervasio, 2008; Markwick and McCammon, 2011; Ovchinnikov and Karplus, 2012 and references therein).

Resistance-Nodulation-Cell Division eﬄux systems of Gram-negative bacteria form tripartite complexes. The inner-membrane RND transporter and a membrane fusion (adaptor) protein (MFP) connect to a channel that traverses the OM called the outer membrane factor or channel (OMF; Ma et al., 1993; Thanassi et al., 1997; Zgurskaya and Nikaido, 1999; Murakami et al., 2002). Some OMFs such as TolC in *E. coli* are highly versatile and involved in the eﬄux of both antibiotics and proteins as part of different eﬄux systems (Akama et al., 2004a; Koronakis et al., 2004; Pietras et al., 2008; Phan et al., 2010; Hinchliffe et al., 2013; Krishnamoorthy et al., 2013). The MFP is suggested to stabilize the assembly of the pump, to contribute to the transfer of eﬄux-coupled conformational transitions from the RND transporter to the OMF and to affect substrate recognition (Ma et al., 1993; Akama et al., 2004b; Mikolosko et al., 2006). X-ray structures of the individual components of AcrAB-TolC and MexAB-OprM (the two main RND eﬄux systems of *E. coli* and *P. aeruginosa*, respectively) have been solved by X-ray crystallography, and also computational studies addressing structural and dynamical aspects of these components have been reported in the literature (Vaccaro et al., 2006, 2008; Schulz and Kleinekathöfer, 2009; Schulz et al., 2010, 2011, 2015; Fischer and Kandt, 2011, 2012; Vargiu et al., 2011; Raunest and Kandt, 2012; Wang et al., 2012; Eicher et al., 2014; Fischer et al., 2014; Blair et al., 2015a). Still open are crucial questions about structure and stoichiometry of the functional assembly (Bavro et al., 2008; Krishnamoorthy et al., 2008; Misra and Bavro, 2009; Symmons et al., 2009; Tikhonova et al., 2011; Xu et al., 2011; Ferrandez et al., 2012; Hinchliffe et al., 2013; Du et al., 2014), and also computational studies on the assembly are still in their infancy and limited to static aspects, not least because of the size and complexity of the whole systems (Symmons et al., 2009; Phillips and Gnanakaran, 2015).

The RND inner-membrane transporter subunit is integral to the function of the system. The transporter subunit of the RND-type tripartite complex functions as a proton/drug homotrimeric antiporter and is key for energy transduction and substrate specificity of the entire three-component complex (Murakami, 2008). Structural data have mainly been collected for AcrB, of *E. coli*, and to a lesser extent for MexB, the homolog of AcrB in *P. aeruginosa* (for a recent review, see Ruggerone et al., 2013a). According to crystallographic results, the shape of the protein resembles that of a jellyfish. Viewed orthogonally to the membrane plane, each protomer elongates for ∼120 Å, comprising a TM region of ∼50 Å composed of 12 α-helices (TM1 to TM12), and a periplasmic headpiece of about 70 Å, the latter being divided into a pore (porter) region and an upper region close to the lower part of the OMF (Murakami, 2008; Eicher et al., 2009).

After the first symmetric structure was solved (Murakami et al., 2002) other crystal structures of AcrB revealed asymmetric conformations of the three monomers in the trimer (Murakami et al., 2006; Seeger et al., 2006; Sennhauser et al., 2007). The three conformations were proposed to represent three consecutive states in a three-step peristaltic mechanism of the substrate translocation [called functional rotation (Murakami et al., 2006) or peristaltic motion (Seeger et al., 2006)]. The postulated functional rotation starts with recognition of substrate at a low affinity site on the L monomer, namely the AP. Global conformational transition converts the L to a T conformation, accompanied by tight binding of the substrate in a designated high-affinity binding pocket, i.e., the DP. Successively, a second peristaltic motion leads to a switch from the T to an O conformation, resulting in the release of the substrate toward the OMF. After substrate release, the O conformation relaxes back to the L conformation restarting the cyclic event. The conversion from the T to the O conformation is suggested to be the major energy-requiring step and should be accompanied by proton binding at the proton translocation site in the TM region. Proton release may occur during conversion from O to L. Computational studies based on targeted MD simulations supported this mechanism and the zipper-like closure of the DP (Schulz et al., 2010).

A 3.0 Å crystal structure of MexB showed the same overall fold as its close homolog AcrB did (Sennhauser et al., 2009). The three monomers constituting MexB assumed an asymmetric conformation supporting the general transport model for this family of multi-drug transporters derived from the AcrB structures (Murakami et al., 2006; Seeger et al., 2006; Sennhauser et al., 2007). However, the conformation of monomer L in MexB showed significant differences at the periplasmic portal to AcrB. In particular, no access to the binding cavity was observed in this subunit. A clear rationale for this structural difference is missing to date. Sennhauser et al. (2009) proposed, among other things, that the differences might be attributed to the L monomer being trapped in an intermediate conformational state between the extrusion and the binding of the substrates. Alternatively, specific resting states of MexB and of AcrB may account for the observed differences. Recently, Nakashima et al. (2013) solved the structure of MexB in complex with a pyridopyrimidine derivative. This work provided the first structural information on MexB-inhibitor interactions. The authors also crystallized free MexB and found a structure largely similar to that described by Sennhauser et al. (2009). The binding geometry of the pyridopyrimidine derivative to AcrB was also resolved, and showed relevant variations in the conformation of the ligand. These findings highlighted that subtle differences in the mechanisms of drug binding and translocation are relevant for the two pumps.

Several key features of the MexB structure, in particular the AP and DP sites, could be mapped onto the structure of AcrB (Murakami et al., 2006; Nakashima et al., 2011; Eicher et al., 2012). The first and until now the only computational study of antibiotic-MexB interactions was based on these data (Collu et al., 2012b). Collu et al. (2012b) investigated the behavior of two carbapenems, meropenem and imipenem, in the AP and the DP. The two structurally related compounds are differently affected by the RND eﬄux pumps. The activity of meropenem is significantly reduced by MexAB-OprM eﬄux whereas the activity of imipenem is not (Masuda and Ohya, 1992; Pai et al., 2001; Pournaras et al., 2005; Walsh and Amyes, 2007). The different sensitivities to RND eﬄux make imipenem and meropenem attractive candidates for a comparative study of carbapenem- eﬄux-pump interactions.

Lacking a crystal structure of MexB in complex with the two molecules, the starting configurations for all-atom MD simulations were extracted from docking runs with the ATTRACT package (May and Zacharias, 2008; May et al., 2008; de Vries and Zacharias, 2012). The systems obtained after solvation and equilibration were simulated for 50 ns. A stronger preference of meropenem for the DP than for the AP resulted from the simulations (binding free energies of -8.1 and 2.4 kcal mol^-1^, respectively). Imipenem had nearly the same low affinity for both pockets (0.6 and 0.4 kcal mol^-1^, respectively). This result agreed with microbiological data showing a fourfold to eightfold increase of the MIC of meropenem but no significant change of the imipenem MIC upon overexpression of MexB in *P. aeruginosa* (Eguchi et al., 2007; Ong et al., 2007; Livermore, 2009; Riera et al., 2011). The contacts between meropenem and the DP extracted from the trajectories were qualitatively consistent with the recently determined structure of a MexB-inhibitor complex (Nakashima et al., 2013), suggesting a reliable prediction of the binding structures by the computational protocol of Collu et al. (2012b).

The AP is probably the first internal site to be occupied by compounds during the binding process. At the AP, imipenem and meropenem pointed their β-lactam rings toward the periplasmic region and the entrance to the DP, respectively. These could be considered as fingerprints of the different behavior of the two compounds. Meropenem tended to move toward the DP, following the plausible extrusion route, while imipenem had a propensity to reach regions close to the periplasm. Interactions with solvent molecules can be extracted and quantified from the trajectories as a further interesting detail in support of this picture (Sterpone et al., 2001; Collu et al., 2012a). Collu et al. (2012a) found that imipenem, unlike meropenem, formed long-lifetime interactions with water molecules inside of MexB.

The docking poses of two compounds in the DP were similar but the 50 ns-long simulations led to different equilibrium binding modes. Meropenem moved in the DP of MexB toward the external channel, assuming a location suitable for extrusion. Imipenem slid away from the entrance to the channel connecting the DP to the extrusion mouth and into a position similar to that assumed by doxorubicin in MD simulations of mutated AcrB F610A (Vargiu et al., 2011). Indeed, Vargiu et al. (2011) found that doxorubicin moved deeper into the DP of a F610A mutant and was not extruded by the induced functional rotation. This was in accordance with reduced doxorubicin MIC (i.e., increased activity) against the F610A variant of AcrB (Bohnert et al., 2008).

The calculated pump interaction patterns have been associated with the different physicochemical properties of the ligand molecules. With its bulky and hydrophobic tail, meropenem established a strong interaction pattern to the aromatic-hydrophobic environment of the DP. Conversely, the more flexible and hydrophilic tail of imipenem had a lower affinity for the DP. Solvent interactions played a major role in the different transport properties of the two carbapenems as well. In fact, the compounds remained highly solvated at all explored binding sites (Collu et al., 2012b). Nonetheless, the water dynamics around meropenem were significantly different in the DP than in the bulk solvent. On the other hand, imipenem showed the same solvent interactions in the DP as in the bulk solvent.

## Paths for Substrate Entry

**FIGURE 3:**
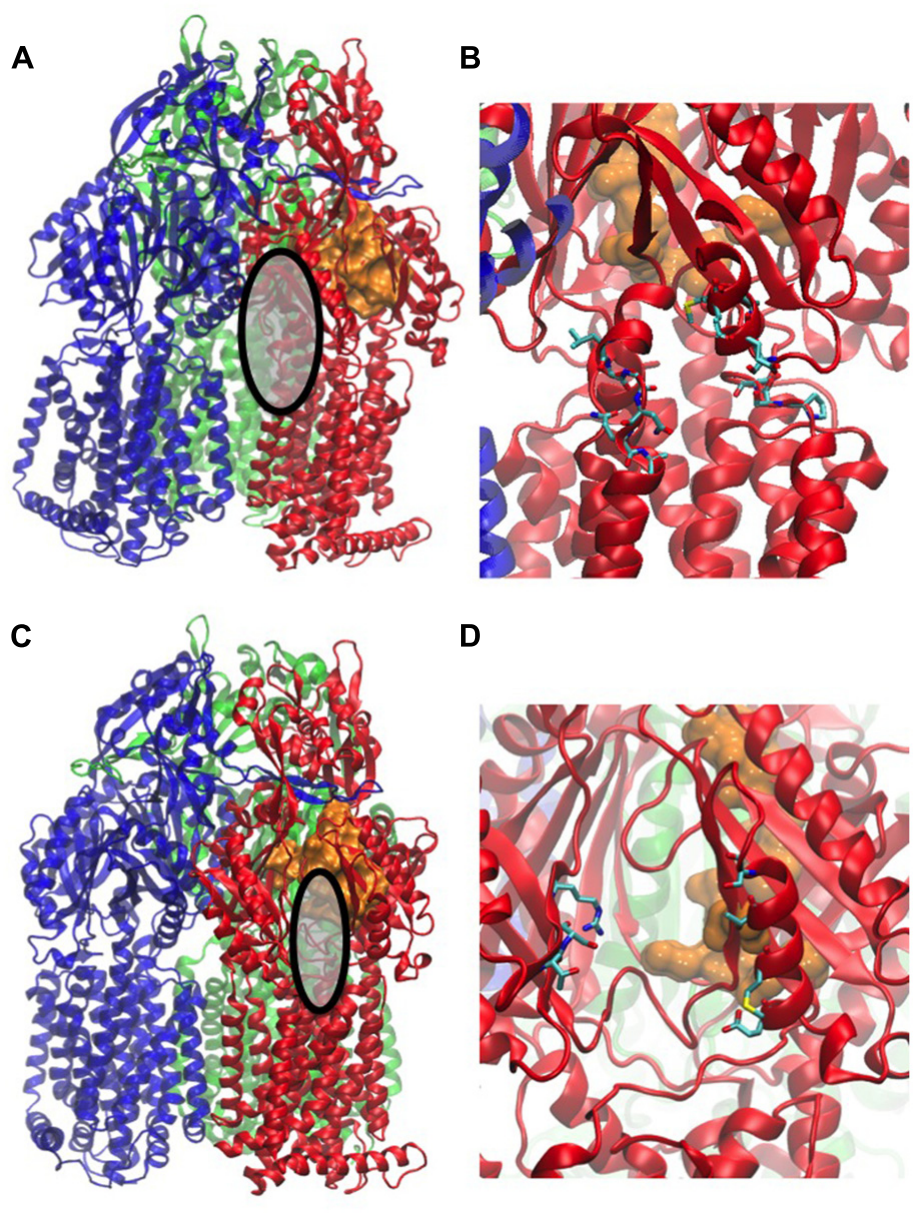
**Main entrances of paths for compounds**. The structure used in the figure corresponds to the PDB code 2V50 (Sennhauser et al., 2009). MexB monomers are colored in blue, red, and green, respectively. The distal binding pocket in the T monomer is represented as orange surface. The entrance of the vestibule is highlighted as gray surface in **(A)** and shown with the atomic details (in licorice colored by atom type) in **(B)**, that of the cleft as gray surface in **(C)**, and its atomic details in **(D)**. All the atomic-level figures were rendered using VMD (Humphrey et al., 1996).

Substrate uptake by RND pumps is still largely unexplored and to our knowledge, only one computational study has addressed this issue (Yao et al., 2013). The entry of substrates into AcrB was studied by a combination of *in silico* tools and site-directed mutagenesis. The study indicated that uptake pathways of minocycline, acriflavine, and novobiocin differed significantly. Novobiocin is the largest and acriflavin the smallest among the three compounds while minocycline is more hydrophilic than the nearly equally hydrophobic novobiocin and acriflavin. All three molecules are typical substrates of AcrB but they vary in molecular size and hydrophobicity. A ligand-dependent drug uptake mechanism was proposed based on the analysis of the free energy associated with drug movement along AcrB tunnels. Strongly hydrophobic and lipophilic drugs of similar size were preferentially taken up via the vestibule path (the entrance of the vestibule path is shown in **Figures 3A,B**). This path starts close to the membrane surface at a region between two protomers and goes via the AP to the DP. Other drugs were translocated through the cleft path starting at a large external indentation of the periplasmic domain, formed by the subdomains PC1 and PC2 of a single AcrB protomer. The cleft path, whose entrance is shown in **Figures 3C,D**, directly connects the periplasm to the DP (Husain and Nikaido, 2010). Smaller drugs were found to favor the vestibule path, while larger compounds preferentially took the cleft path.

The direct simulations identified a novel alternative uptake path, which is not visible in the crystal structure. This third path goes along the bottom of the porter domain toward PC1 and could be validated by site-directed mutagenesis of AcrB in *E. coli*. Mutations of residues located along the path significantly impaired the eﬄux efficiency. The work of Yao et al. (2013) is of great interest because it combined different techniques to gain insight into an important step of the eﬄux process. However, it has to be noted that the authors made several approximations. As in all the computational studies described in this review, the partners of the RND transporter (i.e., AcrA and TolC in the present case) were missing. It was also assumed that the drug uptake was mainly driven by hydrophobic interactions. Finally, some of the physicochemical properties such as the ordering of amphiphilic drugs in the lipid bilayer and the conformational flexibility of drugs with rotatable bonds were neglected. Despite of the necessary approximations, the work of Yao et al. (2013) was an important contribution to the understanding of drug RND eﬄux. The structures of the L monomers and ligand conformations in co-crystals suggest interesting mechanistic differences between MexB and AcrB (Sennhauser et al., 2009; Nakashima et al., 2013). Thus, a similar study on MexB would be of great interest.

## Conclusion

Much progress has been made in the understanding of the transport mechanism of RND eﬄux pumps since they were discovered. Genetic and biochemical investigations have provided a good survey of the substrate specificity of different pump complexes in various bacteria. It has become accepted that RND pumps evolved as a first line of bacterial defense against exogenous substances, allowing the development of additional defense strategies (Olivares et al., 2013). Molecules that are able to penetrate the OM are potentially problematic for Gram-negative bacteria. Many sophisticated studies have supported this view as they have indicated that amphiphilic molecules (such as many antibiotics), which are able to pass the OM, are good substrates for export systems (Nikaido, 2011; Nikaido and Pages, 2012). X-ray crystallography has provided highly detailed structural information on binding interactions between pump subunits and substrates (Ruggerone et al., 2013a). It is intriguing that only a handful of a large number of known eﬄux substrates could be co-crystallized with eﬄux pumps. Although X-ray structures provide snap-shots of a complex transport mechanism, they have been crucial for the setup of a transport model. Concerning specific experiments aiming at a more direct investigation of eﬄux dynamics, studies with whole bacteria have been performed. Whole cell eﬄux assays require thorough controls to rule out non-specific effects that interfere with transport measurements. Such control measurements include the use of membrane-interacting probes to monitor effects on membrane integrity or on the electrochemical potential across the inner membrane. The finding that many of the membrane-interacting probes are eﬄux substrates too, has confirmed the concept of recognition and export of membrane-active compounds. Based on experimental data, new *in silico* tools have been developed and brought to a point where they can reliably describe or even predict the dynamics of compound extrusion by RND pump subunits.

Substrate recognition by RND eﬄux pumps is a multi-factorial process that can be measured by different methods as described in this article. MexB, for example, has a broad substrate spectrum including large (e.g., erythromycin) and charged (e.g., aztreonam, PAβN) molecules (see **Table 1**). MexB transports hydrophobic dyes, many of which have been primarily used to study membrane properties, which indicates a preference of this pump for hydrophobic substrates. This is in agreement with the finding that the activity of the hydrophilic antibiotic ceftobiprole is not significantly affected by MexB. Crystal structures and MD simulations of MexB strongly suggested that the DP, lined with hydrophobic amino acid residues, is the main structural element for the recognition of hydrophobic elements (Collu et al., 2012b; Nakashima et al., 2013). However, hydrophobicity alone is not sufficient to explain substrate recognition by MexB. Imipenem and meropenem have a similar size and hydrophobicity (**Table 1**) but only meropenem is transported by MexB. A computational study revealed that imipenem, unlike meropenem, made no high affinity contacts to the AP or to the DP but instead made long-life interactions with solvent molecules inside MexB and eventually did not enter MexB (Collu et al., 2012b).

The challenge for future studies will be to combine the precision of X-ray structures with the functional relevance of whole cell studies. MD simulations based on experimental data provide a promising tool to complement, integrate and rationalize these data and to shed some light onto this problem.

Despite the advances outlined in the review, dissecting the molecular and conformational steps that regulate transport of substrates by RND pumps is still a very challenging and intriguing task and requires a very efficient interplay between techniques and approaches coming from different fields. The fate of a compound, governed by the action of an RND transporter (i.e., efficiently or poorly transported), is determined by the subtle balance of different molecular contributions that are not necessary large. The interaction of meropenem and imipenem with MexB described in the Section *Computational Study* is a good example: small differences in flexibility and hydrophobicity are suggested to make the former a good substrate and the latter a poor one. Computational methods can offer insight at a level of accuracy that is not reachable by a single experimental technique but they need a continuous feedback from experiments. Further crystallographic studies of RND pumps in complex with substrates and more accurate modeling techniques with extended simulation times for large proteins are needed in order to achieve full atomic pictures of the entire tripartite complexes. Eﬄux kinetics might be affected by several factors that have to be included in the simulations but are not known *a priori* (Kinana et al., 2013). Computer simulations can integrate data from experiments on the molecular level and help to interpret data from whole cell assays (i.e., functional eﬄux pump ensembles).

The development of novel antibiotics that can bypass the effects of MDR pumps or the development of clinically useful EPI is still a challenging task. Understanding the mechanistic details and the structure-function relationship of bacterial eﬄux systems, as well as their regulation and the synergistic interactions between the pumps and other resistance mechanisms, is not only scientifically rewarding but can also stimulate applied research for effective new antibacterial drugs.

## Addendum in Proof

While our manuscript was under review two comprehensive review articles by Li et al. (2015) and by Yamaguchi et al. (2015) were published that cover many aspects discussed in our article.

## Conflict of Interest Statement

Jürg Dreier is employee of Basilea Pharmaceutica International Ltd. Paolo Ruggerone declares that the research was conducted in the absence of any commercial or financial relationships that could be construed as a potential conflict of interest.
